# 2010 International consensus algorithm for the diagnosis, therapy and management of hereditary angioedema

**DOI:** 10.1186/1710-1492-6-24

**Published:** 2010-07-28

**Authors:** Tom Bowen, Marco Cicardi, Henriette Farkas, Konrad Bork, Hilary J Longhurst, Bruce Zuraw, Emel Aygoeren-Pürsün, Timothy Craig, Karen Binkley, Jacques Hebert, Bruce Ritchie, Laurence Bouillet, Stephen Betschel, Della Cogar, John Dean, Ramachand Devaraj, Azza Hamed, Palinder Kamra, Paul K Keith, Gina Lacuesta, Eric Leith, Harriet Lyons, Sean Mace, Barbara Mako, Doris Neurath, Man-Chiu Poon, Georges-Etienne Rivard, Robert Schellenberg, Dereth Rowan, Anne Rowe, Donald Stark, Smeeksha Sur, Ellie Tsai, Richard Warrington, Susan Waserman, Rohan Ameratunga, Jonathan Bernstein, Janne Björkander, Kristylea Brosz, John Brosz, Anette Bygum, Teresa Caballero, Mike Frank, George Fust, George Harmat, Amin Kanani, Wolfhart Kreuz, Marcel Levi, Henry Li, Inmaculada Martinez-Saguer, Dumitru Moldovan, Istvan Nagy, Erik W Nielsen, Patrik Nordenfelt, Avner Reshef, Eva Rusicke, Sarah Smith-Foltz, Peter Späth, Lilian Varga, Zhi Yu Xiang

**Affiliations:** 1 Departments of Medicine and Paediatrics, University of Calgary, Calgary, Alberta, Canada; 2 Department of Internal Medicine, Universita degli Studi di Milano, Ospedale L. Sacco, Milan, Italy; 3 3rd Department of Internal Medicine, Faculty of Medicine, Semmelweis University, Budapest, Hungary; 4 Department of Dermatology, University Hospital of the Johannes Gutenberg-University of Mainz, Mainz, Germany; 5 Department of Immunology, Barts and the London NHS Trust, London, England, UK; 6 University of California, San Diego, San Diego, California, USA; 7 Johann Wolfgang Goethe University, Frankfurt/Main, Germany; 8 Departments of Medicine and Pediatrics, Penn State University, Hershey, Pennsylvania, USA; 9 Department of Medicine, University of Toronto, Toronto, Canada; 10 Department of Medicine, Laval University, Quebec City, Quebec, Canada; 11 Departments of Medicine and Medical Oncology, University of Alberta, Edmonton, Alberta, Canada; 12 Department of Medicine, CHU de Grenoble, Grenoble, France; 13 Member, Patient Advisory Committee, Canadian Hereditary Angioedema Network (CHAEN)/Réseau Canadien d'angioédème héréditaire (RCAH). 705 South Tower, 3031 Hospital Dr. NW, Calgary, Alberta, Canada; 14 Portage La Prairie, Manitoba, Canada; 15 Department of Pediatrics, University of British Columbia, Vancouver, British Columbia, Canada; 16 Department of Medicine, Regina, Saskatchewan, Canada; 17 Memorial University and Janeway Child Health Centre, St. John's, Newfoundland, Canada; 18 Department of Medicine, McMaster University, Hamilton, Ontario, Canada; 19 Department of Medicine, Dalhousie University, Halifax, Nova Scotia, Canada; 20 Department of Medicine, University of Toronto, Oakville, Ontario, Canada; 21 Ancaster, Ontario, Canada; 22 St. Catharines, Ontario, Canada; Member and Chair, Patient Advisory Committee, Canadian Hereditary Angioedema Network (CHAEN)/Réseau Canadien d'angioédème héréditaire (RCAH; 23 Transfusion Medicine, Ottawa Hospital, Ottawa, Ontario, Canada; 24 Department of Medicine, University of Calgary, Calgary, Alberta, Canada; 25 Department of Pediatrics, CHU Sainte-Justine, University of Montreal, Montreal, Quebec, Canada; 26 Department of Medicine, University of British Columbia, Vancouver, British Columbia, Canada; 27 Halifax, Nova Scotia, Canada; 28 Brampton, Ontario, Canada; 29 Queen's University, Kingston, Ontario, Canada; 30 Department of Medicine, University of Manitoba, Winnipeg, Manitoba, Canada; 31 University of Auckland, Auckland, New Zealand; 32 Department of Internal Medicine, University of Cincinnati, Cincinnati, Ohio, USA; 33 Department of Clinical and Experimental Medicine, County Hospital Ryhov, Jönköping, Sweden; 34 Calgary, Alberta, Canada; 35 Department of Dermatology and Allergy Centre, Odense University Hospital, Denmark; 36 Hospital La Paz Health Research Institute, Madrid, Spain; 37 Duke University Medical Center, Durham, North Carolina, USA; 38 Heim Pal Pediatric Hospital, Budapest, Hungary; 39 Dept of Medicine, Academic Medical Center, Amsterdam Area, Netherlands; 40 Institute for Asthma & Allergy, Wheaton and Chevy Chase, Maryland, USA; 41 4th Medical Clinic, University of Medicine and Pharmacy, Tirgu Mures, Romania; 42 Hungarian Association of Angioedema Patients, Budapest, Hungary; 43 Nordland Hospital, Bodo, University of Tromso, Norway; 44 Department of Medicine, County Hospital Ryhov, Jonkoping, Sweden; 45 Tel Hashomer, and Sackler Faculty of Medicine, Tel Aviv University, Ramat Aviv, Israel; 46 Asociación Española de Angioedema Familiar por Deficiencia del inhibidor de C1 (AEDAF), Madrid, Spain; 47 Institute of Pharmacology, University of Bern, Switzerland; 48 Peking Union Medical College Hospital, Beijing, China

## Abstract

**Background:**

We published the Canadian 2003 International Consensus Algorithm for the Diagnosis, Therapy, and Management of Hereditary Angioedema (HAE; C1 inhibitor [C1-INH] deficiency) and updated this as Hereditary angioedema: a current state-of-the-art review: Canadian Hungarian 2007 International Consensus Algorithm for the Diagnosis, Therapy, and Management of Hereditary Angioedema.

**Objective:**

To update the International Consensus Algorithm for the Diagnosis, Therapy and Management of Hereditary Angioedema (circa 2010).

**Methods:**

The Canadian Hereditary Angioedema Network (CHAEN)/Réseau Canadien d'angioédème héréditaire (RCAH) http://www.haecanada.com and cosponsors University of Calgary and the Canadian Society of Allergy and Clinical Immunology (with an unrestricted educational grant from CSL Behring) held our third Conference May 15th to 16th, 2010 in Toronto Canada to update our consensus approach. The Consensus document was reviewed at the meeting and then circulated for review.

**Results:**

This manuscript is the 2010 International Consensus Algorithm for the Diagnosis, Therapy and Management of Hereditary Angioedema that resulted from that conference.

**Conclusions:**

Consensus approach is only an interim guide to a complex disorder such as HAE and should be replaced as soon as possible with large phase III and IV clinical trials, meta analyses, and using data base registry validation of approaches including quality of life and cost benefit analyses, followed by large head-to-head clinical trials and then evidence-based guidelines and standards for HAE disease management.

## Introduction

When our first consensus meeting took place in Toronto, Canada in October 2003, there were no licensed drugs in North America for the treatment of HAE attacks and only two randomized clinical trials with plasma-derived C1 inhibitor replacement therapy (pdC1INH;[1,2]) and a few clinical trials using androgens and antifibrinolytics [3-5]. C1-esterase inhibitor concentrates (Berinert P^® ^and Cetor^®^) were available mostly in Europe at the time [Henkel G. CSL Behring - Personal communication: Berinert approved for HAE acute swelling therapy by Country and year of approval: Argentina 2003; Australia January 2010; Austria 1990; Belgium 2009; Bulgaria 2008; Canada 2010; Cyprus 2009; Czech Republic 2009; Denmark 2009; Finland 2009; France 2009; Germany - 1979 (predecessor product, pasteurized product since 1985); Great Britain 2009; Greece 2009; Hungary 1997; Italy 2010; Japan 1990; Luxembourg 2010; Netherlands 2009; Norway 2009; Poland 2009; Portugal 2009; Romania 2009; Slovakia 2009; Slovenia 2009; Spain 2009; Sweden 2009; Switzerland 1993; USA 2009]. There are now several phase III clinical trials underway or reported in HAE therapy and these have led to the licensing of pdC1INH in many parts of the world including Europe and the United States, bradykinin receptor antagonist Icatibant in Europe, and kallikrein inhibitor Ecallantide in the United States. More phase III clinical trials are currently underway or pending reporting including pdC1INH (Berinert^®^, CSL Behring; Cinryze^®^, ViroPharma; Cetor-n^®^, Sanquin), recombinant C1-INH replacement therapy (conestat alfa; Rhucin^®^, Pharming), kallikrein inhibitor (Ecallantide, Kalbitor^®^, Dyax), and bradykinin-2-receptor antagonist (Icatibant, Firazyr^®^, Jerini/Shire) (reviewed in [6]). Consensus approaches require timely updating and validation and hopefully with the establishment of data base registries for HAE such as the European HAE Register http://www.haeregister.org, the US Hereditary Angioedema Association registry: http://www.hereditaryangioedema.com/, and the European Society for Immunedeficiencies registry http://www.esid.org/esid_registry.php such validation will occur including quality of life (QOL) and cost benefit analyses and drug-drug comparisons. Consensus documents need replacing with evidence-based recommendations based on large phase III and IV trials, head-to-head drug comparisons, meta analyses, guidelines and then standards and we look forward to the improved care of HAE patients as these roll out. To update our previous consensus approach, the Canadian Hereditary Angioedema Network (CHAEN)/Réseau Canadien d'angioédème héréditaire (RCAH) http://www.haecanada.com and cosponsors University of Calgary and the Canadian Society of Allergy and Clinical Immunology (with an unrestricted educational grant from CSL Behring) held our third Consensus Conference May 15th to 16th, 2010 in Toronto Canada. This manuscript is the 2010 International Consensus Algorithm for the Diagnosis, Therapy and Management of Hereditary Angioedema that was agreed to at that conference and this was further circulated for review and comment to previous consensus participants. Speakers at the Conference were encouraged to submit their views for publication and these manuscripts are published together as a thematic publication grouping on HAE in the official journal of the Canadian Society of Allergy and Clinical Immunology: *Allergy Asthma Clinical Immunology*; 2010 (in press [6-16]).

## Patient Group Perspective

Similar to the six Hungarian-sponsored HAE Workshops as indicated in their publication [17], it is appropriate that Patient Groups participate in HAE management consensus discussions to share the patient perspective of HAE management and to help reflect on the development of comprehensive care clinics, home therapy programs, and overall management of HAE. The Canadian and Canadian Hungarian consensus document processes [18,19] included Patient Group participation in discussion, approval, and co-authoring. Patient groups should participate in and coauthor consensus treatment documents affecting their care. The Patient Advisory Committee of the Canadian Hereditary Angioedema Network (CHAEN)/Réseau Canadien d'angioédème héréditaire (RCAH) http://www.haecanada.com and HAE - International Patient Organization for C1 Inhibitor Deficiencies (HAEi) http://www.haei.org participated in the Conference.

## HAE Diagnosis Algorithm: See Figure 1

**Figure 1:**
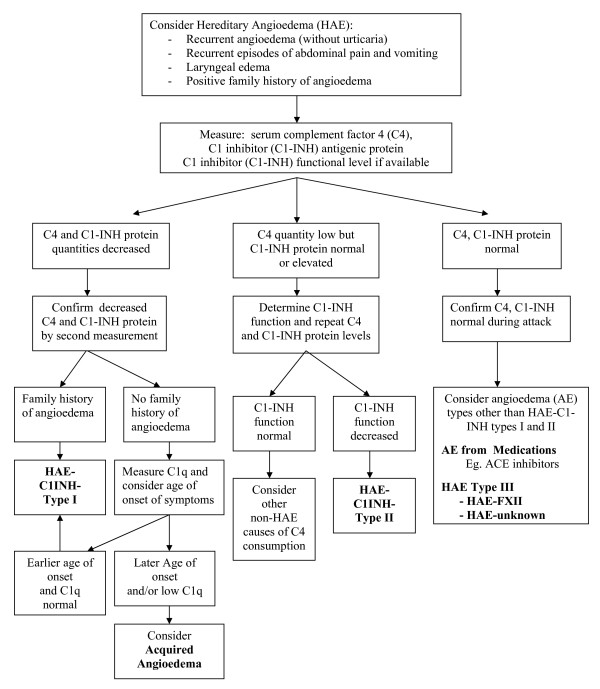
**Hereditary Angioedema - HAE - Diagnostic Algorithm**.

### Clinical Characteristics

Clinical characteristics are reviewed in previous documents [1,6-20]. Patients with HAE may experience recurrent nonpruritic edema of skin and submucosal tissues associated with pain syndromes, nausea, vomiting, diarrhea, and life-threatening airway swellings. Risk of dying from airway obstruction if left untreated is significant [9,17,21]. A prodromal serpiginous erythematous rash is sometimes seen but pruritic urticaria usually makes the diagnosis of HAE unlikely [17,20,22]. HAE genetics are autosomal dominant with 25% spontaneous mutation; the HAE-C1INH gene mapping to chromosome 11q12-q13.1 [17-19]; and the protein defect described by Donaldson in 1963 [23]. An acquired form (acquired angioedema, AAE) was described in 1972 (reviewed in [10]) and is not the focus of this article. AAE differs from HAE having absent family history, late onset of symptoms, usually low C1q antigen levels, prophylactic response to antifibrinolytics often better than to androgens, and sometimes requiring markedly higher doses of pdC1INH with rapid C1-INH catabolism and may respond to Icatibant or Ecallantide [10,24]. Drug-induced angioedema (e.g. angiotensin-converting enzyme inhibitors, ACE-I) is also not included in this discussion [22]. The incidence of HAE is approximately 1:50,000 with no ethnic group differences [17,19]. There seems to be little or no genotype phenotype correlation [17]. Two forms of HAE have been described: type I HAE with low C1-INH antigenic protein and functional activity (85% of cases) and type II HAE with normal or elevated protein but low C1-INH function (15% of cases); and HAE with normal C1-INH often referred to as type III HAE. HAE with normal C1-INH occurs mainly in women and includes HAE associated with mutations in the coagulation factor XII gene and other defects yet to be identified [11,13,18,19,25]. The pathophysiology of types I and II HAE has been elucidated with the candidate molecule resulting in angioedema being bradykinin [17,18,23,25-28]. Age of onset is variable and may present under one year of age [1,6,7,18,19,25] with laryngeal attacks uncommon before age three and tend to occur later than other symptoms [8,18-20,29-31]. Angioedema events often worsen with puberty, estrogen-containing birth control pills, or hormone replacement therapy [8,11,13,15,17,19,20,31,32]. Untreated attacks typically last over 48 to 96 hours [17,20]. Attack triggers may include stress, infections, ACE-inhibitors, minor trauma, menstruation, pregnancy, oral contraceptives but are often unidentified with attacks varying from periodic, clustering, periods of remission [17-20,26,29,31]. Angioedema attacks do not respond to treatment with glucocorticoids or antihistamines, and epinephrine has only a transient and modest benefit [18,19,26,33].

## Diagnostic Algorithm: See Figure 1

Indications for testing include clinical suspicion or positive family history [8,19,20,22,29-31]. Testing under one year of age may not be reliable and should be confirmed after age one (false negative and false positive tests may occur unless using genetic typing) [8,19,20,29-31]. If clinical suspicion of C1-INH deficiency, we recommend screening with C4, (C4 is normal between swelling events in only 2% of cases; [19,31]), C1 inhibitor antigenic protein and C1 inhibitor function, if available. However, a **normal C4 particularly during an edema attack should make one question the diagnosis of HAE **(there is no indication for screening CH50 nor C3) [6,8,10,19,22,29,31]. If serum C4 and C1-INH antigenic proteins are both low (below manufacturer's normal range) and AAE not suspected, then the diagnosis is compatible with **HAE-C1INH-Type I (Type I HAE) **(suggest repeat testing once to confirm). If AAE is possible such as with no family history and later onset of symptoms (age over 40), then serum C1q antigenic protein testing is suggested. If low C1q, the diagnosis is compatible with **AAE **(C1q antigenic protein is reduced in 75% of AAE but usually normal in HAE; [10]). If C4 is normal or low and C1-INH antigenic protein normal but clinical suspicion is strong, HAE is **NOT **ruled out and C1-INH functional assay should be obtained (in a laboratory skilled in functional C1-INH assay with careful sample drawing, handling, shipping, and interpreting results) [8,18,19,28,29,34]. If C1-INH functional activity is low with normal or elevated C1-INH antigenic protein and normal C1q, this is compatible with **HAE-C1INH-Type II (Type II HAE) **(tests should be repeated at least once to confirm the diagnosis; sample mishandling is common) [8,18,19,28,29,34]. If C4 antigenic protein and C1-INH functional assays are both normal, this rules out Types I and II HAE but does not rule out **type III HAE (HAE-FXII and HAE-Unknown) **(normal C1-INH protein and function occurring mainly in women; some with mutations in the coagulation factor XII gene or other unidentified defects; [11,13,19,25,35] nor **medication-related angioedema **(e.g. ACE-I-related Angioedema; [10,19,22]). If C4 and C1-INH protein are normal, we suggest repeating these during an acute attack [19,28]. Genetic testing is usually not necessary to confirm the diagnosis of HAE-C1INH types I and II particularly if positive family history (autosomal dominant with approximately 25% representing de novo mutations) [8,19,29,31]. However, genetic testing is occasionally helpful in confirming HAE-C1INH (particularly before one year of age and cord blood; [8]) and may contribute to investigation of type III HAE [8,11,13,19,25]. Although C4 and C1-INH protein antigen are routine laboratory tests, C1-INH functional assays are specialized laboratory tests and should only be done in reference laboratories with careful attention to sample handling for complement [8,11,13,17,19,28,29,34]. C1-INH functional assays may use chromogenic or C1s binding ELISA assays. Both distinguish between normal and abnormal but the C1s ELISA assay performance may be poor if manufacturer's normal range (> 67%) is used. The reference laboratory should determine normal range locally with receiver operator characteristic (ROC) analysis, since higher cutoff (84%) may give better discrimination [34].

## Baseline laboratory testing at diagnosis at any age and follow up

Baseline blood borne pathogen surveillance (hemovigilance) samples should be collected and stored at baseline and annually including testing for hepatitis B, C, G; HIV; HTLV; parvovirus and future testing for possible emerging pathogens (serum and nucleic acid storage [19,29,31]. As pdC1INH may be required at any time on an emergency basis after diagnosis, hemovigilance and baseline chemistries and urinalysis are best done at diagnosis. Although production methods for pdC1INH may differ, safety of new generation pdC1INH has been excellent [19,28,29,31,36-38]. Since attenuated androgens may predispose to lipid abnormalities [39] and liver disorders including liver cancer, we suggest [1,7,11,12,17,19,37] serum lipid profile and liver function tests be obtained prior to androgen administration and abdominal liver and spleen ultrasound be performed prior to continuous androgen administration (repeated annually) [8,17,19,28,29,31,40]. Liver function studies (including alanine aminotransferase, ALT, total bilirubin, alkaline phosphatase, albumin, alk phos, and possibly PT/PTT and alpha fetoprotein); creatine kinase (CK), lactic dehydrogenase (LDH), blood urea nitrogen (BUN), creatinine (Cr), complete blood count (CBC) and differential; as well, urinalysis should be obtained at diagnosis [8,17,19,28,29,31,40,41].

## Vaccination recommendations

We recommend that patients at risk for receiving blood products receive vaccination to hepatitis B (may be combination hepatitis A) [8,19,29,31].

## Medications to avoid in patients with HAE

Some medications may trigger or worsen angioedema events in patients with HAE and should be avoided including estrogen contraceptives, hormone replacement therapy, and ACE-Inhibitors [8,11,13,17,19,22,29,31,32]. Plasminogen activators are a theoretical risk but the benefit may outweigh the risk [19].

## Short-Term Prophylaxis - see Figure 2

**Figure 2:**
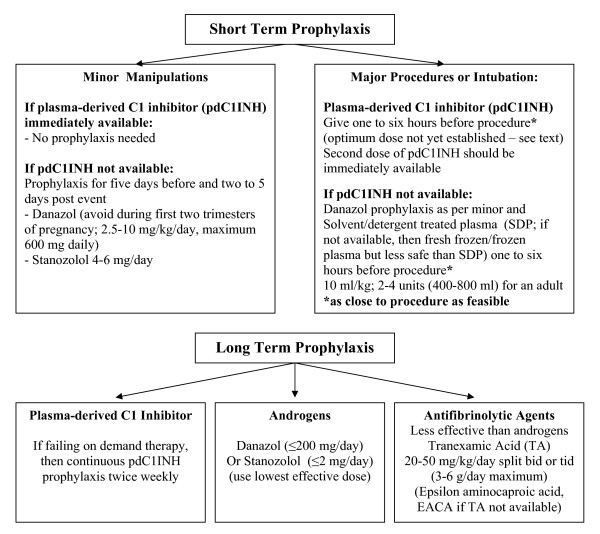
**Hereditary Angioedema - HAE - Prophylaxis Algorithm**.

**Minor Manipulation - s**uch as mild dental work (injection of local anaesthetic may precipitate an attack): if pdC1INH is immediately available, then no prophylaxis (unless such manipulations have previously precipitated an attack in that patient in which case prophylaxis with pdC1INH should be considered). If pdC1INH is not available, then 17-alpha-alkylated anabolic androgen (Danazol most widely used but also stanozolol and oxandralone) or antifibrinolytic prophylaxis (if available, tranexamic acid is preferred to epsilon aminocaproic acid) (see Figure 2). Tranexamic acid as a 5% mouthwash may decrease bleeding from dental procedures and may prevent bradykinin formation in plasminogen rich saliva [42-44]. If considering more than mild manipulation, pdC1INH prophylaxis should be considered. If pdC1INH not available, then short term Danazol is recommended (even in children and last trimester of pregnancy - avoid in the first two trimesters of pregnancy; [8,15,19,29,31,32]. The recommended dose is 2.5 to 10 mg/kg/day, maximum 600 mg daily, for five days before and two to five days after the event [8,19,29,31]; Stanozolol 4-6 mg/day is an alternative [29]. **Whenever possible, PdC1INH should be immediately available **[8,19,29,31]. Since anabolic androgens such as Danazol are more efficacious in the short term compared to antifibrinolytics such as Tranexamic acid (TA; Cyklokapron^®^) or epsilon aminocaproic acid (EACA; Amicar^®^), anabolic steroids are more often used for short term prophylaxis in the setting where pdC1INH is not available [8,19,29,31]. The recommended dose for oral TA (not fully established) is 25 mg/kg two to three times daily with maximum 3 to 6 g daily; IV dose 10 mg/kg two to three times daily adjusting the dose for renal impairment [19,29,31,33,45-48].

**Intubation or major procedures - pdC1INH **one hour pre surgery - as close to procedure as feasible - less than six hours before the procedure (should always be given if endotracheal intubation or manipulation; [8,9,12,14,19,29,31,45]. The optimal dose for prophylaxis for procedures has not yet been established - we recommend 10 to 20 units per kg [8,9,12,14,19,29,31,45]. A second dose of equal amount should be immediately available at time of surgery. Repeat daily as needed until there is no further risk of angioedema. If pdC1INH is not available, then Danazol or Stanozolol are recommended as in V.1 (see figure 2; androgens preferred to TA; TA in doses as above; [19,29,31,33,45,47].) Solvent/detergent treated plasma (SDP; 10 ml/kg; 2 to 4 units, 400 to 800 ml per adult infusion) is an option one to six hours presurgery (fresh frozen plasma or frozen plasma is less safe than SDP; [8,9,19,28,29,31,33,48]; Dr. Mike Frank's group has reported using two units fresh frozen plasma the night before, [49-51]).

### Pregnancy

pdC1INH prophylaxis is the safest prophylactic agent during pregnancy [12,15,19,29,31,32]; discussed at the 6^th ^International HAE Conference held in Budapest in June 2009; dose as in V.2).

### Pediatrics

except when undergoing surgical or diagnostic interventions in the head and neck region, short-term prophylaxis is less often required in children than adults (dosing as in V.1 and V.2; [8,31]).

## Long-Term Prophylaxis: See Figure 2

Prophylaxis indications have been reviewed [12,17,19,52]. Consider prophylaxis with antifibrinolytics, attenuated androgens, or pdC1INH if more than one severe event per month occurs and if a treatment for acute attacks is not sufficiently effective or is not available [8,12,19,28,37,52-54]. It should be noted that: **the number of events per year does not predict severity of the next event nor whether the first or next event will be an airway event**.

### 17-alpha-alkylated anabolic androgens

Attenuated androgens such as Danazol and Stanozolol are the usual agents with methyltestosterone and oxandrolone as alternatives. Androgens are generally more effective than antifibrinolytic agents [8,17-19,40]. Androgen contraindications usually include pregnancy, lactation, cancer, hepatitis, and childhood (until finished growing) [8,15,17-19,29,31,32]. Side effects may include virilization, weight gain, acne, hair growth, altered libido, voice deepening, decreased breast size, menstrual irregularities, vasomotor symptoms, hypertension, atherogenesis, altered lipid metabolism, altered liver enzymes, cholestasis, hepatic necrosis, liver neoplasms (hepatocellular adenomas or carcinomas), erythrocytosis, hemorrhagic cystitis, and ambiguous genitalia in newborns if mothers treated with androgens during pregnancy [8,17,19,28,37,40,41,55,56]. Androgen induction can be with high dose and reduce or low dose and escalate aiming to achieve the lowest effective dose (maximum long term doses recommended are 200 mg daily for Danazol and 2 mg daily for Stanozolol) [17-19,28,29,31,35,37,40,55]. Androgen therapy is not recommended for children but has been used in the prepubertal setting [8,17,19,29,31,35]. If patients are exposed to a precipitating factor such as infection or if the sensation of prodromal attack symptoms or mild clinical manifestations developing, then doubling the dose for several days has been tried. The lowest effective maintenance dose including trying alternate day or twice weekly should be tried [19,28,29]. Danazol has been used in children [8,31,35] but pdC1INH may be the safest long term approach [8,31,35]. Danazol has been used for prophylaxis in HAE type III as have progesterone and tranexamic acid [11].

#### Androgen Monitoring

every six months: liver enzymes (ALT, AST, alk phos), lipid profile, complete blood cell count, and urinalysis. For adults with a dose of 200 mg or less per day Danazol: suggest an annual liver spleen ultrasound. In prepubertal patients or in adults with doses higher than 200 mg Danazol daily: suggest six monthly liver spleen ultrasound for the detection of focal lesions and annual alpha fetoprotein [8,19,29,31,35,57-60].

#### Antifibrinolytic Agents (AFs;[45])

Tranexamic acid (TA; Cyklokapron^®^) is more effective than epsilon aminocaproic acid (EACA; Amicar^®^;[3]) and has mostly replaced EACA outside the USA. AFs may not be as effective as androgen therapy in HAE but may be useful in AAE [10,17,19,28]. TA is mostly used for prophylaxis in children before Tanner V puberty stage or if not wanting to risk androgen prophylaxis [8,17,19,29,31,35]. Dyspepsia is common and can be reduced by taking the drug with food. Other side effects may include myalgia, muscle weakness, elevated serum creatine phosphokinase or aldolase, rhabdomyolysis (EACA particularly), hypotension, fatigue, and retinal changes (seen in animals) [19,35,44,45]. TA dosage is not well established [4,8,17,19,29,31,35,45] aiming for the lowest effective maintenance with recommended starting dose of 20 to 50 mg/kg/day (split 2 to 3 times daily, taken with food, with daily maximum of 4 to 6 g; [4,8,17,19,35,44,45]. The dose may be able to be reduced to 0.5 g once or twice daily or even alternate-day or twice weekly regimens [29]. **TA Monitoring: **six monthly CK, urinalysis, liver and renal function; annual ophthalmology check for eye pressure (risk of glaucoma) [8,19,29,31,35]. AFs have not been associated with excess thrombosis or myocardial infarction in controlled trials [61-64], but there are case reports of thrombosis in patients with hypercoagulable states treated with AFs [65,66], so it is prudent to use it cautiously if there is a family history of thrombophilia or active thromboembolic disease [35,45,65,66]. TA was reported effective long-term prophylaxis in HAE type III [67].

### Plasma-derived C1 inhibitor - pdC1INH

Home pdC1INH self-infusion programs should be offered to patients (created similar to hemophilia self-infusion programs which have existed for 35 years; [8,12,17,19,29,31,36,37,52,68,69]. The dose including dose per kg for prophylaxis has not been fully established [12,14,36,37,70]. We recommend 500 units (if less than 50 kg, 110 lb) or 1000 units (if greater than 50 kg, 110 lb) [1,12,14,19].

**Cinryze^® ^**from ViroPharma is FDA approved for adolescent and adult prophylaxis at a dose of 1000 units every three or four days (see FDA approved package insert:

http://www.fda.gov/BiologicsBloodVaccines/BloodBloodProducts/ApprovedProducts/LicensedProductsBLAs/FractionatedPlasmaProducts/ucm150480.htm)[12]. Prophylaxis with pdC1INH is not 100% effective http://www.cinryze.com/documents/cinryze-prescribing-information.pdf. Cetor^® ^from Sanquin is licensed in the Netherlands http://www.sanquinreagents.com/sanquin-eng/sqn_products_plasma.nsf/8551110e498bd2c8c12572110034decf/11343072be4286d2c125702a004a4e50/$FILE/Cetor%20SPC.pdf.

**Berinert^® ^**from CSL Behring is approved for therapy in many countries around the world including Europe and by USA FDA (see FDA approved package insert:

http://www.fda.gov/BiologicsBloodVaccines/BloodBloodProducts/ApprovedProducts/LicensedProductsBLAs/FractionatedPlasmaProducts/ucm186264.htm). Reconstitution and administration of PdC1INH as per package inserts (see above web links; [18,19,35]). **DO NOT SHAKE **as this will denature the protein. Administration should be via peripheral vein (usually over ten minutes) (see product package insert references above for administration details) [8,18,19,29,31,35].

## Treatment of Acute HAE Attacks - see Figure 3

**Figure 3:**
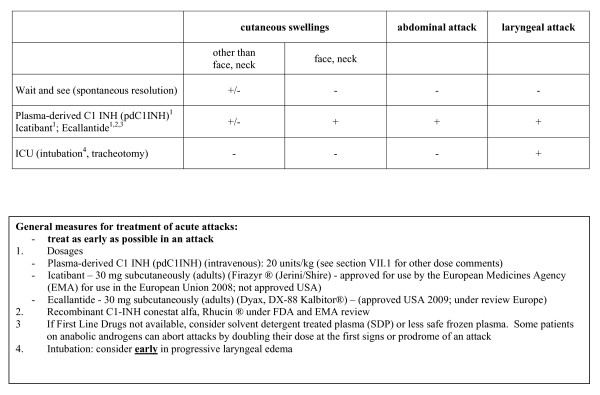
**Treatment of Acute Hereditary Angioedema - HAE - Attacks**.

**We recommend treating attacks as early as possible**.

### Plasma-derived C1-INH - PdC1INH

PdC1INH has been the first line therapy for several decades in Europe and elsewhere and used for many years in Canada under Special Access Program [8,12,17-19,29,31,35,37,38,48,52-54]. **Berinert^® ^**from CSL Behring was licensed by USA FDA October 9^th^, 2009 for therapy of HAE events and licensed in many other countries for many years. **Cetor^® ^**from Sanquin has been available in The Netherlands for some time [35,53]. Berinert^®^, CSL Behring, has been shown to be more effective than placebo for therapy of acute angioedema attacks at a dose of 20 units/kg (see package insert reference above; [12,35,71]). However, use in other countries is 500 to 1500 units [8,14,19,29,31,37,53,54,72]; Cetor dose recommendation is 1000 units - http://www.sanquin.nl/sanquin-eng/sqn_products_plasma.nsf/8551110e498bd2c8c12572110034decf/11343072be4286d2c125702a004a4e50/$FILE/Cetor%20SPC.pdf). PdC1INH has been well tolerated and viral transmission attributed to new generation pdC1INH has not been reported [33,36-38,73]. As pdC1INH is a blood product, annual recipient hemovigilance and vein-to-vein tracking are essential (tracking and hemovigilance similar to home therapy programs for Hemophilia Comprehensive Clinics). PdC1INH has been used to treat HAE attacks in HAE Type III [11,67].

### Icatibant

Icatibant (Firazyr^® ^from Jerini/Shire) is a small peptide, bradykinin receptor blocker approved for use in treatment of HAE in the European Union. Dose is 30 mg subcutaneously in adults. Pediatric experience is pending. Although not usually needed, the dose can be repeated six hourly twice more if needed (see package insert for Firazyr^®^). Local reactions are common with injection [6,28]. Icatibant may be beneficial in type III HAE [11,74].

### Ecallantide

Ecallantide, DX-88, Dyax, Kalbitor^® ^is a small peptide, kallikrein inhibitor approved for treatment of HAE in the USA since December 2009. Dose is 30 mg subcutaneously (adults). It is not recommended for self infusion at this time because of a small risk of anaphylaxis and is being further studied in phase IV clinical trial [6].

### Emerging Therapies

**Recombinant C1-INH**, conestat alfa, Rhucin^® ^is recombinant human C1-INH produced in transgenic rabbit milk [6,28]. Currently under FDA review, in June 2010 it received a positive opinion from the European Medicines Agency's (EMA) Committee for Medicinal Products for Human Use (CHMP) for the treatment of acute angioedema attacks in patients with HAE. With this positive opinion, the CHMP recommends the European Commission to grant the European Marketing Authorization. The product will be marketed in the EU under the name Ruconest^®^.

As new therapies become available, it will be very important to conduct rigorous phase IV clinical trials (utilizing data base registries such as HAEA and HAEI and ESID provide) so that long term safety efficacy data on these therapies can be closely monitored and to allow comparison of cost benefit studies including quality of life issues between the various therapies. This will provide funding organizations and patients better information on which to base their choices of products provided under pharmaceutical plans and the most cost effective product for patient choice. It is exciting to finally have licensed therapeutic and prophylactic medications for treatment of this disorder.

#### Other treatments

If the above currently available therapies such as pdC1INH, Icatibant, and Ecallantide are not available, other therapies may include increasing (usually doubling) the androgen (Danazol or Stanozolol) dose or antifibrinolytics [8,17,19,29,31,35]. However, unlike pdC1INH, Icatibant, and Ecallantide, there are limited data to support this recommendation [3-5]. Use of solvent detergent plasma (SDP; preferred for viral transmission reasons over FP/FFP) could theoretically worsen attacks and remains controversial and again there are no clinical trials to support its use [8,19,29,31,35]. Adrenaline has been used but is usually of only modest and transient benefit [8,19,29,31,75]. Pain management, intravenous fluids, and supportive care are essential but do not affect the outcome of an attack and therefore are not a replacement for early intervention with pdC1INH, Icatibant, Ecallantide or possibly recombinant C1INH or other emerging therapies.

## Comprehensive Care Clinics - Home Therapy: see Appendix 1

Comprehensive care clinics for immunedeficiencies, rare blood disorders, hemophilia, cystic fibrosis, asthma, cancers and many other disorders have improved survival [76,77] and contributed to improved standard of care for these disorders (see proceedings of the Canadian National Rare Blood Disorders meeting:

http://www.hemophilia.ca/en/about-the-chs/collaboration/network-of-rare-blood-disorder-organizations/2009-progress-in-comprehensive-care-for-rare-blood-disorders-conference----presented-by-csl-behring/#c969). Comprehensive care for HAE is based on the recognition that HAE is a chronic disease and care is complex, requiring a highly specialized and multidisciplinary approach. A comprehensive care clinic must provide accountability for in-hospital and home use of expensive and potentially toxic treatments, track outcomes (both beneficial and adverse), and develop and meet Standards of Care for HAE. It is recommended that HAE patients be linked with comprehensive care clinic programs (bringing together clinical care, education and research) to facilitate diagnosis, therapy, management; facilitate data base registries; allow rigorous safety efficacy monitoring of emerging therapies of HAE; and to facilitate access to home therapy programs (similar to the model for comprehensive care of hemophilia) (see blood disorder conference link above; [8,16,19,29,31,36,35,37,54,68]). One clinic model can be found in Appendix 1 (also see [19,29,31]). Patients are encouraged to carry "alert" identification (wallet card example may be found at: http://www.haecanada.com/files/WalletCard_Bilingual.pdf) and an accompanying letter indicating the diagnosis of HAE (with type), materials necessary to be carried for care for presentation at air line and other security areas, and outlining instructions for administration of intervention therapy (such as infusion of pdC1INH). It is recommended that HAE organization websites provide infusion instructions for downloading by patients and comprehensive care clinics (example of home infusion technique may be viewed at: http://haecanada.com/infusion/index.html) Home therapy and particularly home pdC1INH infusion programs should be offered to patients. Such programs should be created similar to hemophilia home infusion programs which have existed for 35 years (see blood disorders link above; [8,14,16,17,19,29,31,35-37,52,54,68]). Home care was discussed at the 6^th ^International HAE Conference held in Budapest in June 2009 http://www.haenet.hu/new/program_C1INH2009.pdf and the resulting home care consensus approach has been assembled [16].

## Pediatrics

Most of the pediatric considerations of HAE are incorporated in the above algorithms (Figures 1, 2 and 3 and Appendix 1) and have been reviewed elsewhere. Most treatment drugs have been licensed for adults with pediatric licensing pending [6,8,14,18,19,29-31,35].

## Pregnancy and Lactation

Most of the pregnancy and lactation considerations of HAE are incorporated in the above algorithms (Figures 1, 2 and 3 and Appendix 1) and have been reviewed elsewhere. Most treatment drugs have not been trialed in pregnancy and are not licensed for use in this setting although there is anecdotal use of pdC1INH use in pregnancy and lactation [6,15,19,29,32,33,52,53]. Tranexamic acid can be found in breast milk [44].

## Conclusion

Since our first Canadian International Consensus meeting in 2003 when plasma-derived C1-inhibitor concentrates had been available for decades in Europe but not widely outside Europe, many new therapies have emerged in HAE management. Many phase III clinical trials have been completed and some reported on. Several products are now licensed for prophylaxis and therapy of HAE and hopefully are reducing the morbidity and mortality in this disorder. These therapies and home care concepts are providing freedom for work, travel, and every day activities including sports activities with more normalization of life style and improved quality of life for HAE patients. We must strive to elevate the standard of care for HAE patients through comprehensive care clinics and home care programs and institute safety, efficacy, and cost benefit monitoring. Data base registries may provide the health care systems, patients, and patient groups with the necessary data to choose the most appropriate individualized management of one's HAE. Consensus approaches are only interim guides to chronic and rare diseases such as HAE and should be replaced as soon as possible with more phase III studies, meta analyses, large phase IV post-marketing trials, and head-to-head studies using data base registry validation of consensus approaches including quality of life and cost-benefit analyses followed by guidelines and then standards for HAE disease management.

## Appendix 1 - Comprehensive Care Clinics for Hereditary Angioedema - 2010 05 27

**(Modified by permission from: **http://www.haecanada.com - comprehensive care clinics)

### Comprehensive Patient Care Clinics: Clinical care, Education, and Research

Comprehensive care for HAE is based on the recognition that HAE is a chronic disease and care is complex, requiring a highly specialized and multidisciplinary approach. A comprehensive care clinic must provide accountability for in-hospital and home use of expensive and potentially toxic treatments, track outcomes (both beneficial and adverse), and develop and meet Standards of Care for HAE.

### Comprehensive HAE Clinics will Provide

1 Best Clinical Treatment outcomes including:

a. a comprehensive care team made up of nurse coordinator, clinician, social worker, data manager, pain management specialist, genetic counsellor, and administrative support;

b. access to specialized diagnostic testing;

c. access to home treatment;

d. a networked Patient Information System to facilitate product recalls - collect data on therapy outcome measures and safety, and facilitate participation in clinical trials

e. access to clinical advances as they become available;

f. access to 24 hour support;

g. access to up-to-date standards of care, including standardized wallet cards;

h. tracking and intermittent audit of quality outcomes including beneficial and adverse outcomes through secure, comprehensive and networked data management.

2 Education of patients and staff regarding:

a. responsible Self/Family Care (home care model) with home and self infusion/administration instruction and support;

b. developments in the cause, diagnosis, treatment, outcomes, and prognosis of HAE

c. changes in the administrative management of the clinic

3 An environment conducive to research including:

a. access to and support for clinical trials of new treatments;

b. access to and support for translational research in diagnosis and prognosis;

c. accesss to and support for psychosocial research such as quality of life studies.

4 An advisory or oversight board with patient group representation for each clinic

## Competing interests

Many of the authors have either entered consultancy with or have been involved in educational programs and their organization, had direct funding from, have been speakers for, or have had consultation agreements with CSL Behring, Dyax, Jerini, Pharming, ViroPharma, Shire. The 2010 International Consensus Algorithm for the Diagnosis, Therapy and Management of Hereditary Angioedema was arrived at during the Canadian Hereditary Angioedema Network (CHAEN)/Réseau Canadien d'angioédème héréditaire (RCAH) second meeting held May 15^th^/16^th^, 2010, Toronto, Canada and was cosponsored by CHAEN/RCAH, the Canadian Society of Allergy and Clinical Immunology, and the University of Calgary and was funded through an unrestricted educational grant from CSL Behring. Publication of this manuscript is sponsored by University of Calgary.

## Authors' contributions

TB prepared the manuscript. All authors have read, revised and approved the manuscript.
